# Indents

**Published:** 1913-07-15

**Authors:** 


### INDENTS.

**☞Brief Articles of Technical or Scientific Value will receive notice and comment. Contributions invited.**

*Failures in Casting.* Many failures in casting are traceable to the use of too large a sprue former. This permits the gold when in a highly molten state to descend a short distance in the large sprue hold and there to become lowered in temperature, thus forming in some cases an obstruction which blocks the passage from the crucible to the mold. So in all ordinary casting it is preferable to use a relatively small sprue former and one that is cylindrical rather than conical in form. R. I. Wood, in *Review.*

*Gold Shell Crowns.* A while ago, in a confrere's laboratory, the subject of making gold shell crowns was on tap. My friend undertook to make one in ten minutes, ready to cement on a root. Taking a model in which was imbedded a natural molar root in place of a missing molar, when the minute hand of his watch was on line he began. He did not rush like a race horse, but took it quietly, like an old cow chewing her cud. He handed me the finished seamless crown, neatly fitting the root, neatly contoured, with well-shaped contact points, and perfectly fitting the occluding teeth, in exactly eight minutes. Now, all that was needed to make it ready for cementing was to flow inside to strengthen the occluding surface about one pennyweight of gold solder, smooth and bevel the gum margin, and to polish it; all of which he could readily do in another eight or ten minutes. He told me it was no uncommon thing for him to bow a patient out with a shell crown in place within half an hour after having the root prepared. He keeps on hand models of shell crowns, in various shapes and sizes, made of pure tin. When the root is prepared he selects one of these, modifies its shape as required, by skillful use of a burnisher, and fits it to the root. Then the patient is directed to chew upon it. It is carefully removed, any breaks repaired, and a fusible metal model made into which a gold thimble is swaged. There is no secret in the process, and it has been a surprise to the writer that this or some other seamless method, is not more generally used. There is no accounting for taste, however, some like the long way round.—W. H. Trueman, in *Brief*.

*Inlay Investment.* A mixture of silex 3 parts and dental plaster one part, by weight, makes an excellent and inexpensive investment compound for cast work. Mix to a creamy consistency, eliminating air bubbles by jarring plaster bowl on bench while stirring. (Never jar ring while pouring up.) When wax has burned out allow investment to cool down before casting, irrespective of casting method employed. This investment will not crack if properly mixed, and the castings will come out with a smooth, brilliant surface. P. G. Puterbaugh, in *Review*.

*Varnish for Plaster Casts.*

| | |
|---|---|
| Ether | 4 oz. |
| Collodion | 2 oz. |
| Silver glass | 2 oz. |

The silver glass may be obtained from dealers in painters' supplies. Keep the solution well corked and allow it to stand forty-eight hours before using, and apply with a camel's-hair brush. It gives the model a nice, white, glossy surface.—Dr. H. R. Lewis, Chicago, *Dental Review*, March, 1913.

*The Business Side of Dentistry.* There is no doubt that professional men, such as lawyers, physicians and dentists, are as a rule poor business men. The very nature of their occupation makes it impossible for them to compete with others engaged in those commercial pursuits commonly classed as "business" occupations; they naturally do not apply to the conduct of their own pursuits the rules and standards necessary for success in the transaction of business. It would, undoubtedly, often be to their advantage to do so. Many of the articles recently published in our journals on this subject contain excellent advice on the application of business methods to the conduct of practice. We feel, however, that many of the entuhsiastic contributors to this department of dental literature have gone too far, and that the subject has, in their hands, degenerated until it has become a "service-selling" proposition, undignified in character and unprofessional in its application to practice, inasmuch as their chief object seems to be to teach a way by which two dollars may be taken from the public where heretofore it has been possible to extract but one.—Editorial, *Pacific Dental Gazette,* March 1913.

*Cleansing and Sterilizing Solution.* Take a strong solution of potassium bichromate and acidulate it with strong sulphuric acid (the commercial is less expensive and is as efficient for this purpose as the more expensive grades), adding the acid a very little at a time, preferably out of doors. Be careful not to inhale the fumes. The resulting solution is a powerful oxidizer and will "cut" almost any deposit from bottles, mixing slabs, etc. It must always be handled with care, as it is destructive to skin, clothing, etc. It is the photographer's universal cleansing solution, attacking energetically all organic matter, and may be repeatedly used before losing its usefulness.—W. H. Trueman, in *Brief.*

*Cement Amalgam Filling.* Place one-half of a bunch of amalgam, when ready to pack, on a slab, and then drop a proportionate amount of cement acid onto the amalgam, then add the cement powder and grind the whole mass as required in mixing the cement to a soft consistency. Immediately fill the cavity with the mixture, trim the edges, then add the rest of the amalgam, mix and finish with clear amalgam. The above method well carried out will be a "specific" that will not need an experimental station. To prove the above, cut out the filling a few years afterwards and be convinced. The cement mixed as described will cut off the thermal or galvanic changes and be comfortable in the mouth.

The time has arrived when filling teeth with gold or amalgam as a saver of teeth can hardly be called first-class work. Professor Brackett says of the adhesive joint: "It is as much better as a well mortared is better than a dry brick wall in masonry."—L. C. Taylor, *in Brief.*

*Care of the Operator's Hands.* In surgical cases, the operator's hands and arms are brushed thoroughly with hot water and tincture of green soap. Particular attention is given to the nails, which are pared and cleansed with a nail file, and orange-wood stick. The hands are then rinsed in sterile water, bathed in alcohol, and immersed for some time in a sublimate solution. If rubber gloves are worn, the hands are dried in a sterile towel and powdered with sterile talcum powder, and the sterile gloves are put on. If there are any cuts or abrasions upon the fingers, these are painted with a coat of collodion. In daily dental practice the thorough brushing of the hands and fingers with a good soap and hot water is sufficient If an operator is treating a syphilitic patient and should injure the skin, the immediate and very thorough massage of the wound with $33\frac{1}{3}$ percent. calomel ointment is a prophylactic measure against infection. The hands must always be very thoroughly dried before going out, to prevent chapping.—W. J. Lederer, *Dental Digest.*

*Taking the Bite.* After all "has been done and said" in favor of mechanical methods of taking the bite, notwithstanding that they have been worked out with scientific accuracy, one wonders if there is any better way than the old fashioned one of adjusting the wax rims on the trial plates until, when in position they "look right." Of course that "look right" means a great deal. The school boy artist and his friends are charmed with his sketch of the family cow, although they may have a lurking thought that it was realy intended to be a portrait of the pig. Many have no artistic ideals, and, on the other hand, there are many patients whose facial make-up violates all rules of artists and dentists, and to whom a correctly articulated full denture proves a useless incumberance, while one that violates all accepted rules may prove a pleasure and delight. The true artist should be familiar with what should be, and yet not so wedded to it that he is unable to see when and where conditions alter cases. What should be has its limitaions. The artistic eye and an educated mechanical sense are the court of last resort. It is a question whether with a "good judge" on the bench it is not more generally reliable than the complicated machine contrivances and instruction books for bite taking, just now so much in evidence.—W. H. Trueman, in *Brief.*

*Preparing a Gold Inlay Wax Pattern for Investment.* When the wax model for a gold inlay has been trimmed to correct form, and the surface has been carefully gone over with a very small quantity of oil of cajuput, it is carefully washed off in water and gone over with liquid soap and water. Besides giving a beautiful finish to the wax, it will be found that this treatment enables the operator to paint the investment on the wax much more readily.—E. S. Best, *Dental Review.*

*Bulk in Porcelain Inlays.* It is quite true that the inlay must have sufficient bulk to be able to withstand the stress placed upon it by mastication. That goes without saying, and there are cases where one sometimes deliberately runs a little risk; but one fact I have established to my own satisfaction is that, under favorable conditions, if one has two millimeters of thickness of a good low-fusing porcelain, such bulk is sufficient to endure the strain of mastication. Of course, one ordinarily takes as much bulk as one can get, but in many delicate cases I am convinced that two millimeters' thickness is sufficient for the integrity of the properly fused porcelain inlay. I should also like to add that there is far less danger of spoiling a low-fusing porcelain than a high fusing one.—N. L. Jenkins, in *Cosmos.*

*Desensitizing Sensitive Dentin.* Hyper-sensitive dentin at the cervical margin is best treated with a concentrated solution of sodium bicarbonate in glycerin thus avoiding discoloration as produced by silver nitrate.—*Odont. Tidskrift,* per *Oesterreich. Zeitschr. f. Sotmatologie.*

*Vinegar for Softening Plaster.* Vinegar is a very good means for removing hardened plaster of Paris from the hands, or from flasks. Plaster models can be easily removed from an articulator by pouring a little vinegar upon the portion where the bow is embedded in plaster.—*Oesterreich. Zeitschr. f. Stomatologie.*

*Gold Filling in Artificial Teeth.* I notice that in the May issue of the *Cosmos,* page 528, a clinic is reported in which the clinician demonstrates a method of putting gold fillings in artificial teeth by means of grooves and cement. I think the following procedure, which I have been using for certainly ten years, to be much better for the purpose.

Grind a small depression where the gold filling is to be placed; cover this with Jenkin's inlay body mixed to a thin cream. Press in the piece of crystal gold, the size of the depression, and fuse in an electric furnace. When cold, more gold can be malleted to the gold base, using the same care as with a gold filling. I have never yet seen one of these fillings come away from the tooth. I trust this will interest your readers.—C. Every Brown, L.D.S., in *Cosmos.*

*Burnishing the Gold Inlay.* Whatever may be the cause or causes, cast inlays do not always fit. They sometimes fit well except at certain points. The readiness with which the cast gold flows under stress, due to a disturbance of its molecular arrangement in the process of casting, greatly facilitates "burnishing," so-called and the same seems to be resorted to in many instances as a means of improving the fit. That good is often accomplished in this way is unquestionably true, but that harm may be—indeed often is—done is also quite true.

When resorted to after the setting of the inlay and the hardening of the cement, as is seemingly so often done, if the burnishing accomplishes anything at all beyond the smoothing of the exposed surface of the gold, it does harm instead of good.

The cement is crushed at the margins, and, imprisoned though it may be for a time, its dissolution and washing out by the secretions and the darkening of the margins are the results which are sure to follow.

Burnish the inlay—burnish it well—but always do so either before the cementation of the inlay or before the setting of the cement.—J. H. Crossland, in *Brief.*

*Advantages of the Porcelain Inlay.* It seems scarcely necessary to recapitulate the advantages of the porcelain inlay, but still I venture to remind you that it is not from the esthetic standpoint alone that it surpasses all others.

It is to the patient the least distressing method of restoration. It requires less loss of structure than is regarded as essential with the gold inlay, and it is exempt from the discomfort often occasioned by a large gold inlay in a vital tooth. From the moment that a porcelain inlay is set, the patient forgets that the tooth containing it was ever defective; neither heat nor cold effect it, and it can be used in eating or drinking or can be brushed with either cold or hot water with impunity. The large gold inlay, despite its film of protecting cement, remains a body which conducts caloric changes.

It is the duty of the dentist not only to restore lost tissue, together with contour and indestructible masticating surface, but also to give his patient complete exemption from subsequent discomfort. This can be permanently accomplished with porcelain alone, and, speaking from the results of wide experience, I wish to add that there is no position in the mouth in which the skilful dentist may not, if he sees fit, successfully place a porcelain inlay which will bear unimpaired the strain of mastication.

The well-made porcelain inlay also renders the tooth practically exempt from the secondary caries. It does not shrink in cooling, as does the cast inlay, and has no edge to be dubiously burnished over defective cervical margins. It may be known before setting whether a porcelain inlay fits, and if it is defective anywhere, it is better to make another rather than hazard an ultimate failure.

But it is in its esthetic aspect that the porcelain inlay is most attractive. It is a great achievement to place a mouth, ravaged by dental caries, in a comfortable and sanitary condition; but to add restoration of the original beauty of the teeth is a supreme triumph, and one worthy of the wonderful age in which we live.

In America the porcelain inlay was born; in Europe it has attained to its highest development. These facts are of much significance—for our profession is no longer provincial, but it is everywhere working upon the same problems in a generous spirit of fraternal rivalry, and for the good of all the world. N. S. Jenkins, in July *Cosmos.*

*Bacterial Metabolism and Internal Medicine.*

Evidence, both qualitative and quantitative, is presented by Kendall, illustrative of the nature and extent to which utilizable carbohydrate spares protein and protein derivitives from bacterial attack. The carbohydrate protects protein largely from bacterial break down.

The sparing action is not complete, but it results in the practical elimination of nitrogenious products, incidental to bacterial development, some of which may be toxic, and the substitution for them of substances which are non-nitrogenous, which may be irritative, but probably never truly toxic. This evidence has been introduced here chiefly to indicate how the principle involved may be made use of theoretically in the treatment of diseases of bacterial origin, where the anatomic features of the disease are such as to permit the utilization of carbohydrate by the invading micro-organisms. The object of the diet rich in carbohydrate is twofold; physiologically to provide the patient with a readily assimilable food requiring a minimal amount of digestive energy to prepare it for the tissue needs, and bacteriologically to shift bacterial metabolism from the destruction of body tissue for their food requirements to the utilization of carbohydrate for at least the major part of their dietary needs. This does not directly result in annihilation of the invading bacteria. but it certainly approaches their metabolic reformation. The shifting of metabolism to sugars appears to deprive these microbes of one of their most potent weapons of offense, and forces them to act on the defensive; forcing the parasite to act on the defensive theoretically at least, permits the host to rally and to strengthen its defensive and even its offensive powers earlier in the battle. The initial damage, brought about when the host is temporarily overwhelmed by the initial onslaught of the invaders (which is accomplished largely during the incubation period of the disease) cannot be estimated at present, and perhaps cannot be undome. The final struggle between host and parasite can be influenced directly, and favorably.—*Journal A. M. A.*, June 21, 1913.

